# The potential of pirtobrutinib in multiple B-cell malignancies

**DOI:** 10.1177/20406207221101697

**Published:** 2022-06-16

**Authors:** Jeffrey L. Jensen, Anthony R. Mato, Camila Pena, Lindsey E. Roeker, Catherine C. Coombs

**Affiliations:** Department of Medicine, The University of North Carolina at Chapel Hill, Chapel Hill, NC, USA; Memorial Sloan Kettering Cancer Center, New York, NY, USA; Memorial Sloan Kettering Cancer Center, New York, NY, USA; Memorial Sloan Kettering Cancer Center, New York, NY, USA; Division of Hematology, Department of Medicine, The University of North Carolina at Chapel Hill, 170 Manning Drive, Chapel Hill, NC 27599, USA

**Keywords:** acalabrutinib, BTK, CLL, ibrutinib, LOXO-305, non-covalent, pirtobrutinib

## Abstract

Bruton’s tyrosine kinase (BTK) is a critical downstream signaling element from the B-cell receptor (BCR) that has been effectively inhibited in B-cell cancers by irreversible, covalent inhibitors including ibrutinib and acalabrutinib. All FDA-approved covalent BTK inhibitors rely on binding to the cysteine 481 (C481) amino acid within the active site of BTK, thus rendering it inert. While covalent BTK inhibitors have been very successful in multiple B-cell malignancies, improving both overall survival and progression-free survival relative to chemoimmunotherapy in phase 3 trials, they can be limited by intolerance and disease progression. Pirtobrutinib is a novel, highly selective, and non-covalent BTK inhibitor that binds independently of C481, and in a recent, first-in-human phase 1/2 clinical trial was shown to be extremely well tolerated and lead to remissions in relapsed/refractory patients with multiple B-cell malignancies. Here, we review the pharmacologic rationale for pursuing non-covalent BTK inhibitors, the clinical need for such inhibitors, existing safety, and resistance mechanism data for pirtobrutinib, and the forthcoming clinical trials that seek to define the clinical utility of pirtobrutinib, which has the potential to fulfill multiple areas of unmet clinical need for patients with B-cell malignancies.

## Biology and pharmacology of BTK inhibition in B-cell cancers:

BTK is a non receptor, cytoplasmic tyrosine kinase in the Src subfamily encoded by the *BTK* gene on the X chromosome.^1,2^ When mutated, the *BTK* gene confers B-cell development deficits with x-linked immunodeficiency in mice and agammaglobulinemia, the virtual absence of all B cells and immunoglobulins in humans. These deficits are thought to be mediated by disruption of downstream signaling from the BCR, which is known to transmit survival and proliferation signals in both healthy and malignant B cells.^3^ Binding of the BCR in healthy B cells results in formation of a ‘signalosome’: A complex of kinases and scaffold proteins tethered to the plasma membrane that initiates a cascade of phosphorylation mediated by the tyrosine kinases LYN, SYK, and subsequently BTK. BTK signaling leads to activation of PLCG2 and production of downstream second messengers inositol-1,4,5-triphosphate and diacylglycerol, activation of protein kinase C, and activation of transcription factors including NF-κB.^2^

BTK inhibition was first established as a valuable therapeutic target for B-cell cancers by ibrutinib: a potent small-molecule inhibitor of BTK that binds covalently to C481 in the active kinase domain of BTK.^1^ In cell culture, it had a modest effect inducing apoptosis but with effective blockade of BCR signaling and the subsequent cytokine production (i.e., CCL3 and CCL4).^4–7^ While ibrutinib binds readily to BTK, it also has off-target effects on at least 19 other kinases including BLK, BMX, ITK, TEC, EGFR, ERBB2, and JAK3. This non-specific activity is at least partially responsible for the side effects of ibrutinib.^8,9^ For example, off-target TEC inhibition is attributed to some of the increased anti-platelet and bleeding activity of ibrutinib.^10^ Acalabrutinib, a second-generation covalent BTK inhibitor that also binds C481, demonstrates much higher potency and selectivity for BTK over other kinases.^8,9^ Similarly, zanubrutinib, another covalent BTK inhibitor, has potential selectivity benefits over ibrutinib. Due to covalent BTK inhibitor resistance mediated by C481 mutations, there was significant interest in the development of BTK inhibitors to target an alternate site as resistance mechanisms overlap for all currently available covalent BTK inhibitors (ibrutinib, acalabrutinib, and zanubrutinib).^11^ Further, covalent BTK inhibitors have short half-lives (e.g., 2–4 h for zanubrutinib) and the serum levels of covalent BTK inhibitors drop below their IC_50_ levels using common dosing regimens possibly enabling tumor progression.^12^ Theoretically, these irreversible inhibitors might be exhausted by BTK turnover, especially in aggressive tumors like Richter’s transformation that are rapidly dividing and have low response rates to covalent BTK inhibitors.

To address mechanisms of resistance and tolerability issues, non-covalent BTK inhibitors have been developed and are at various stages of clinical testing: vecabrutinib, fenebrutinib, nemtabrutinib (MK-1026, formerly ARQ 531), and pirtobrutinib (formerly LOXO-305). Vecabrutinib was studied as monotherapy in a phase 1/2 dose escalation and expansion trial for CLL/SLL or non-Hodgkin’s lymphoma, but this was terminated due to suboptimal activity (NCT03037645).^13^ Fenebrutinib is being studied in autoimmune indications; there are no current clinical trials testing it in hematologic malignancies.^14^ Preliminary results from the phase 2 dose-expansion study of nemtabrutinib in B-cell malignancies were recently presented, which included 51 CLL/SLL patients of whom 43 (84.3%) had prior BTK inhibitor therapy. Nemtabrutinib demonstrated an overall response rate (ORR) of 57.9% for 38 efficacy-evaluable CLL/SLL patients, with grade ⩾ 3 treatment-emergent adverse effects in 68% of total participants (80 of 118).^15^

Pirtobrutinib is a novel third generation, non-covalent BTK inhibitor that inhibits BTK independently of C481 mutations in Waldenstrom’s macroglobulinemia (WM), diffuse large B-cell lymphoma (DLBCL), and CLL cells.^16–18^ It has marked selectivity with more than 300-fold selectivity for BTK over 98% of other kinases with nanomolar potency against both wild-type and C481-mutant BTK in cell and enzyme assays.^16,19,20^ In murine xenograft models with the DLBCL line TDM8, pirtobrutinib exhibited efficacy nearly identical to ibrutinib with BTK wild-type tumor cells but conferred significant improvements in efficacy with BTK-C481S mutants, with nearly half the mass dosing of pirtobrutinib (pirtobrutinib 30 mg/kg BID *versus* ibrutinib 50 mg/kg BID).^21^ In contrast to covalent BTK inhibitors, pirtobrutinib is also able to maintain plasma levels above the BTK IC_90_ with well-tolerated daily dosing.^12,17^

## Clinical successes and limitations of covalent BTK inhibitors

Since the clinical introduction of ibrutinib in 2011, covalent BTK inhibitors have been established as standard of care for a number of B-cell malignancies though an unmet clinical need persists for many patients. Here, we will focus on the role of existing BTK inhibitors for four malignancies: CLL/SLL, mantle cell lymphoma (MCL), WM, and Richter’s transformation (RT) to set the stage for the current areas of unmet need that availability of pirtobrutinib might fill.

### CLL/SLL

After ibrutinib was granted accelerated FDA approval based on phase 2 studies, ibrutinib was tested in randomized phase 3 trials for both relapsed/refractory and untreated CLL and performed admirably with improvements in progression-free survival (PFS) and overall survival (OS). Ibrutinib was superior to ofatumumab in the relapsed/refractory setting,^22^ superior to chlorambucil as frontline treatment,^23^ superior to bendamustine and rituximab as frontline treatment,^24^ and superior to FCR chemotherapy as frontline therapy.^25^ Taken together, these results demonstrated that ibrutinib with or without rituximab was an efficacious therapy and generally superior in both upfront and relapsed/refractory settings to chemoimmunotherapy or anti-CD20 monoclonal antibodies alone.

Next-generation covalent BTK inhibitors have had similar successes in randomized phase 3 CLL trials, both compared with standard-of-care agents and against ibrutinib. Acalabrutinib has shown improved PFS compared with idelalisib and compared with chemoimmunotherapy.^25,26^ Acalabrutinib was also non-inferior to ibrutinib in PFS but with lower rates of atrial fibrillation/flutter and hypertension.^27^ Finally, in the first interim analysis of the ALPINE trial that compared zanubrutinib to ibrutinib, zanubrutinib led to a higher ORR, longer PFS, and a lower rate of atrial fibrillation/flutter.^28^

Covalent BTK inhibitors are commonly discontinued in real-world practice because of toxicities and disease progression. In a series of 616 CLL patients treated with ibrutinib, 41% of patients had discontinued ibrutinib within a median follow-up time of 17 months.^29^ Among those discontinuations, nearly half were secondary to adverse events including atrial fibrillation, infectious complications, and cytopenias. Disease progression accounted for approximately 20% of discontinuations and RT accounted for approximately 5% of discontinuations. The first real-world studies with acalabrutinib (mostly in ibrutinib intolerant patients) show lower discontinuation rates (19%), albeit with shorter follow-up, with a similar proportion discontinuing for toxicity (8 of 13 patients).^30^

Disease progression in CLL through covalent BTK inhibitors is clearly associated with the previously mentioned *BTK-*C481 mutations, but also with *PLCG2* mutations, which can occur in isolation or in conjunction with a BTK mutation.^11,31^ Inhibition of *BTK*-C481 mutated cell growth by BTK inhibitors that do not rely on C481 binding gives strong evidence that *BTK* mutations are causative for the development of resistance.^32^ As PLCG2 is activated by upstream signaling from BTK, *PLCG2* mutations are thought to be activating mutations and have been found more broadly throughout multiple domains of the PLCG2 protein.^33^ Interestingly, certain *PLCG2* mutations are more or less common depending on the co-occurrence of *BTK* mutations, implying cooperation between *BTK* and *PLCG2* in overcoming ibrutinib therapy.^33^

Among patients progressing on BTK inhibitors, options are limited though venetoclax has shown success in both clinical trials and using real-world evidence.^34–36^ PI3 K inhibitors including idelalisib and duvelisib were studied prospectively in BTK inhibitor naïve patients^37,38^ and have limited success (median PFS 7 months) in patients who have previously progressed on a covalent BTK inhibitor in real-world studies.^39^ Rates of allogeneic stem cell transplants for CLL have been decreasing in the United States and in Europe since the introduction of BTK inhibitors, but high-risk CLL patients that are suitable transplant candidates and are failed by covalent BTK inhibitors and venetoclax have limited treatment options and still undergo transplant in modern practice.^40–42^ However, most refractory CLL patients are elderly and not considered transplant candidates. Patients who have previously been exposed to both BTK inhibitors and venetoclax represent a major area of unmet need. In the largest real-world study to date of ‘double-exposed’ CLL patients (prior treatment with venetoclax and covalent BTK inhibitors), PFS depended on the next line of therapy. Median PFS was 5 months for PI3 K inhibitors, 11 months for allogeneic stem cell transplant, 4 months for CAR T-cell therapy, and only 3 months for chemoimmunotherapy but among 40 patients that received a non-covalent BTK inhibitor the median PFS was not reached (median follow-up of 9 months).^43^ In another study that did not include non-covalent BTK inhibitors, patients that progressed through a covalent BTK inhibitor and through venetoclax had a dismal OS of 3.6 months.^44^ In summary, while covalent BTK inhibitors are mainstay therapy for both upfront and relapsed/refractory CLL, there are limited therapeutic options for patients failed by a covalent BTK and venetoclax due to either CLL progression, drug toxicities, or RT.

### Mantle cell lymphoma

MCL is an incurable B-cell lymphoma with poor long-term outcomes. Generally treated with chemoimmunotherapy with or without autologous stem cell transplant upfront in fit patients, covalent BTK inhibitors are reserved for frontline treatment in frail patients or in the relapsed/refractory MCL setting.^45^ BTK inhibitors were introduced in the relapsed/refractory setting after a phase 2 trial showed ibrutinib to have durable single-agent efficacy.^46^ A phase 3 trial comparing ibrutinib to temsirolimus demonstrated an improved ORR, PFS, tolerability, and a trend toward an OS benefit for ibrutinib.^47,48^ Ibrutinib has also been used in combination with venetoclax and in combination with lenalidomide and rituximab in phase 2 trials.^49,50^ Both acalabrutinib and zanubrutinib received FDA approval for relapsed MCL based on phase 2 monotherapy trials with median PFS of 19.5 months and 21.1 months, respectively.^51–53^ Recent real-world studies have confirmed similar lengths of PFS for patients with relapsed MCL treated with BTK inhibitors.^54^ *BTK*-C481 mutations are less commonly observed among MCL patients progressing on covalent BTK inhibitors, where *TP53* alterations occur in 75% of cases.^55^

Patients have few therapeutic options following progression on a covalent BTK inhibitor; chimeric antigen receptor (CAR)-T cells are one approach but these are limited to patients with access to tertiary referral centers.^56^ Retrospective studies demonstrate short OS following progression on ibrutinib, ranging from 2.9 to 5.5 months.^57,58^ Patients with MCL are also similarly susceptible to the toxicities of non-covalent BTK inhibitors including cytopenias, infections, atrial fibrillation, and bleeding.^59^

### Waldenstrom’s macroglobulinemia

WM is a rare B-cell lymphoma characterized by elevated serum levels of IgM and proliferation of IgM-producing lymphoplasmacytic cells. Owing to the rarity of WM, there have been few phase 3 randomized studies conducted. iNNOVATE was a randomized, double-blind, placebo-controlled phase 3 study of ibrutinib or placebo in combination with rituximab in patients with either treatment-naïve or relapsed WM.^60^ PFS at 30 months was 82% among the ibrutinib-rituximab treatment arm and 28% in the placebo-rituximab arm (*p* < 0.001). This effect was independent of *MYD88* and *CXCR4* mutational status and persisted in subgroup analyses of treatment-naïve or relapsed WM patients. These compelling results led ibrutinib to become the first FDA-approved therapy for WM. More recently, the ASPEN trial, a head-to-head, phase 3 trial comparing ibrutinib and zanubrutinib showed a similar complete response (CR) and very good partial response (VGPR) rates (28% *versus* 19% for zanubrutinib and ibrutinib, respectively with *p* = 0.09), but zanubrutinib was better tolerated with lower rates of atrial fibrillation and other toxicities.^61^ The ORR for zanubrutinib-treated patients improved with 3 years of follow-up of the original phase 1/2 trial (ORR 95.9%, VGPR/CR 45.2%).^62^ Ibrutinib and chemoimmunotherapy are mainstays of therapy in both the treatment naïve and relapsed/refractory setting with venetoclax and protaseome inhibitor regimens reserved for BTK inhibitor relapse.^63^ Mechanisms of relapse in the setting of WM include C481 and PLCG2 mutations.^64^

### Richter’s transformation

RT, especially when clonally related to the underlying CLL, carries extremely poor outcomes and inadequate treatment strategies. A variety of chemoimmunotherapy regimens have been utilized such as R-CHOP (rituximab, cyclophosphamide, doxorubicin, vincristine, and prednisone), R-EPOCH (rituximab, etoposide, prednisone, vincristine, cyclophosphamide, and doxorubicin), and OFAR (oxaliplatin, fludarabine, ara-C, and rituximab) though with low CR rates and short PFS.^65–67^ Responses to ibrutinib and acalabrutinib for relapsed/refractory RT have been demonstrated in small series, though the duration of response is typically short lived (3–6 months median duration response).^68,69^ Moreover, as BTK inhibitors have become frontline therapy for CLL in both previously untreated and relapsed/refractory CLL patients, the proportion of covalent BTK inhibitor naïve patients have decreased which may further decrease the response rate upon transformation.^70^

## Clinical evaluation of pirtobrutinib to date

The first-in-human clinical trial of pirtobrutinib was a multicenter phase 1/2 study (BRUIN) in patients with CLL, MCL, WM, and other B-cell lymphomas. Eligible patients were exposed to either one to two prior lines of therapy (CLL/SLL eligible after frontline covalent BTK inhibitor after fifth study amendment) with endpoints of maximum tolerated dose (MTD) (phase 1) and ORR (phase 2).^17,21^ Pirtobrutinib was well tolerated at all dose levels, with no dose-limiting toxicities. Subsequently, an MTD was not reached, and 200 mg daily was selected as the recommended phase 2 dose. Atrial fibrillation occurred in less than 1% of patients, which was considered unrelated to pirtobrutinib due to a history of previous atrial fibrillation in affected patients. Hemorrhage, another common covalent BTK inhibitor toxicity, was seen in less than 5% of patients, with only one grade 3 event due to a subarachnoid hemorrhage sustained in a bicycle accident. The most common adverse events were fatigue (20%), diarrhea (17%), contusions (13%), and neutropenia (13%). 87% of adverse events were grade 1 or 2, and the most common grade ⩾ 3 adverse event was neutropenia, with neutropenia reflecting the only grade 4 adverse event. Upper respiratory infections were the most common infection, seen in 7% of patients.^17^

At time of initial publication, 323 patients with a variety of B-cell malignancies have been treated. Efficacy varied among tumor type. ORR was 63%, 52%, and 68% among CLL/SLL, MCL, and WM, respectively. Similar response rates were seen in limited cohorts for follicular lymphoma (*N* = 8, ORR 50%) and in RT (*N* = 8, ORR = 75%). Within CLL/SLL, the ORR and the quality of responses were found to increase with time even in a heavily pre-treated cohort of patients, and a remarkably high proportion of patients (88%) remained on pirtobrutinib with a median follow-up period of ~ 6 months. Response rates were similar between all patients and those previously treated with BTK inhibitors in CLL/SLL (63% and 62%, respectively), MCL (52% for both), and WM (68% and 69%, respectively). In both CLL and MCL, highly refractory patients who had previously received cellular therapies including CAR-T cells and stem cell transplants (autologous and allogeneic) were among the responders. As expected, patients harboring *BTK* C481 mutations were also among the responders. In fact, CLL/SLL patients previously treated with a covalent BTK inhibitor with a *BTK* C481 mutation had similar response rates to those who had wild-type *BTK*. Response rates also seemed unaffected by *TP53* mutation status, 17p deletion, or both.^17^

An expanded dataset for patients with RT was subsequently presented, demonstrating the efficacy of this drug in this otherwise difficult to treat disease.^71^ The drug was tolerated in the RT population with similar rates of adverse reactions as to those in the study at large. ORR among DLBCL patients were lower than for other B-cell lymphomas, with only six of 25 (24%) patients with DLBCL having a response. Of those six who did respond, three only stayed on pirtobrutinib for 3 months and then discontinued. The other three remained on treatment at the time of publication with treatment times of 2.4, 7.1, and 9.6 months. This was in a heavily pre-treated cohort, with the 25 patients having 3–5 prior lines of therapy (median = 4).^17^

## The future of pirtobrutinib

Several clinical trials, summarized in Table 1, are ongoing to establish the role for pirtobrutinib in the treatment of B-cell malignancies. NCT04666038 (BRUIN CLL-321) randomizes patients to pirtobrutinib or investigator’s choice of either idelalisib plus rituximab or bendamustine plus rituximab and requires previous treatment with a covalent BTK inhibitor. Notably, prior venetoclax therapy is not required for enrollment on this trial. If positive, this trial could lead to approval of pirtobrutinib in the post-covalent BTKi setting, which would allow for an additional therapeutic option for patients who have either contraindications to venetoclax therapy such as significant renal disease or inability to complete the required dose ramp necessary for tumor lysis syndrome monitoring. In addition, availability of pirtobrutinib could also help fill an existing major area of unmet need in CLL—an effective therapy for post-covalent BTKi and post-venetoclax treated patients.^44^ Real-world data cited previously already suggests that in ‘double-exposed’ patients, treatment with a non-covalent BTK inhibitor leads to longer PFS than PI3 K inhibitors or chemoimmunotherapy.^43^

**Table 1.** Ongoing phase 3 clinical trials with pirtobrutinib.

Trial	Population	Experimental Arm	Control Arm
NCT05023980, phase 3	Untreated CLL/SLL	Pirtobrutinib	Bendamustine + Rituximab
NCT04965493, phase 3	Previously treated CLL/SLL	Pirtobrutinib + Venetoclax + Rituximab	Venetoclax + Rituximab
NCT04666038, phase 3	BTK inhibitor pre-treated CLL/SLL	Pirtobrutinib	Investigator’s choice of Idelalisib + Rituximab or Bendamustine + Rituximab
NCT04662255, phase 3	Previously treated, BTK inhibitor naïve MCL	Pirtobrutinib	Investigator choice of covalent BTK Inhibitor

BTK, Bruton’s tyrosine kinase; CLL/SLL, chronic lymphocytic leukemia/small lymphocytic lymphoma; MCL, mantle cell lymphoma.

NCT04965493 (BRUIN CLL-322) investigates the addition of fixed-duration pirtobrutinib to venetoclax and rituximab compared with venetoclax and rituximab alone in patients with at least 1 prior line of therapy (not required to be a covalent BTK inhibitor). This study seeks to improve on the existing efficacy of the MURANO regimen of venetoclax and rituximab, which leads to a median PFS of 53.6 months.^72,73^ Few patients in the original MURANO trial had prior BTK inhibitors, though by design one would expect ~80% patients in this trial to have had prior covalent BTK inhibition, so the median PFS may differ even in the control arm. Should pirtobrutinib improve the efficacy of venetoclax and rituximab alone, this may become an attractive therapeutic option especially for younger, fitter patients seeking a time-limited therapy option.

NCT05023980 (BRUIN CLL-313) investigates pirtobrutinib as frontline therapy for CLL/SLL compared with bendamustine and rituximab. Similar to other trials comparing continuous BTK inhibition in the frontline setting to the bendamustine and rituximab regimen,^24,74^ it is plausible that this will be a positive trial and may then allow for the approval of pirtobrutinib in the frontline setting. If the tolerability profile remains similar, then pirtobrutinib could lead to significant improvements in patient outcomes given the known high rate of discontinuation of other covalent inhibitors.^29^

In MCL, NCT04662255 is currently recruiting patients and investigates previously treated MCL patients that are BTK inhibitor-naïve. Patients will be randomized to receive either pirtobrutinib or the investigator’s choice of the three FDA-approved covalent BTK inhibitors, with a primary endpoint of PFS. Should pirtobrutinib lead to superior PFS, this may lead to improved outcomes among MCL patients needing BTK inhibitor therapy. This study will also provide valuable information regarding the head-to-head toxicity profiles of pirtobrutinib with covalent BTK inhibitors.

Multiple lines of evidence in cell culture, xenografts, and clinical trials suggest that pirtobrutinib can effectively treat C481-mutated tumor clones.^21^ Recently published data based on the sequencing of heavily pre-treated CLL patients before and after developing pirtobrutinib resistance have implicated non-C481, *BTK* kinase domain mutations (V416 L, A428D, M437R, T474I, and L528 W) and *PLCG2* mutations as resistance mechanisms for pirtobrutinib.^75^ The numbers in this initial study are small, only evaluating nine patients with pirtobrutinib resistance, but all nine pirtobrutinib-resistant patients had *BTK* kinase domain mutations or *PLCG2* mutations. Cell line studies were also consistent with these kinase domain mutations conferring both non-covalent and covalent BTK inhibitor resistance, raising the concern that pirtobrutinib-refractory patients might largely be refractory to covalent BTK inhibitors in the covalent BTK inhibitor-naïve setting, but it is unknown if patients treated with pirtobrutinib earlier in their disease course will develop these same mechanisms of genetic escape. It will also need to be determined if patients with WM, MCL, or RT develop these same mutations.

## Conclusion

Inhibiting BTK has yielded remarkable gains for patients with B-cell malignancies, and the development of pirtobrutinib furthers these gains by introducing a new treatment option for patients with CLL/SLL, MCL, and other lymphomas. Pirtobrutinib circumvents C481 mutations as a genetic mechanism of resistance for covalent BTK inhibitors, extending the utility of BTK inhibition in the management of these diseases by creating an additional line of therapy for patients with covalent BTK-inhibitor-refractory disease. Pirtobrutinib is also highly selective for BTK, with resultant tolerability leading to few discontinuations for adverse events,^17^ and thus far has favorably low rates of atrial fibrillation when compared with rates seen with covalent BTK inhibitors.^27^ While the majority of ongoing trials are focusing on CLL/SLL, forthcoming data in MCL may also establish a role for pirtobrutinib in the relapsed/refractory disease setting where progress is greatly needed. In addition, there may be a role for pirtobrutinib in other aggressive B-cell cancers including RT, diseases for which advances are needed given such poor outcomes observed with existing therapies. Pirtobrutinib further builds on the success of covalent BTK inhibitors, demonstrating activity in patients with BTK-inhibitor-refractory disease and safety with an excellent adverse event profile. Multiple trials are ongoing, which will further inform the optimal use of pirtobrutinib, and FDA approval of pirtobrutinib is anticipated
